# Medical Institutions of Italy

**Published:** 1846-07

**Authors:** Wm. W. Morland

**Affiliations:** Milan


### MEDICAL INSTITUTIONS OF ITALY.

*Milan*, May 18, 1846.

To the Editor of the Boston Medical and Surgical Journal.

*Dear Sir*,—Although it may possibly prove a superfluous undertaking, (so well are the European institutions known,) I have thought it might not be unacceptable to you to run over a few notes upon the hospitals of Italy, to several of which I have made a point of going, although not always having sufficient time to go over them in a thorough manner.

Italy certainly possesses many very noble institutions for the poor and the sick—and was, during the middle ages, far in advance of the rest of Europe. At Naples, the "Royal Poor House" should be mentioned as an institution of great usefulness and merit. It was commenced in 1751, and now is an immense building; one side is allotted to females, and the other to males. At present, between 5 and 6000 (according to the statements) are maintained and instructed by this establishment: among other things the inmates are taught *surgery*. The hospital for *Incurables* is capable of containing upwards of 1000 persons; the sick are received from all parts of the kingdom—and foreigners also. There are cliniques also—medicine, surgery, midwifery, an anatomical theatre, &c.

In Rome, the hospitals are not so well looking, *internally*, nor, I should think, so well conducted, as in most of the other Italian cities. The small hospital of Benfratelli, containing 80 beds, is much neater and better ventilated than San Spirito, the principal one. The Benfratelli is in the hands of the monks, who perform the services and duties for the sick. The aspect of things was exceedingly *dubious* as to the comfort of the patients. The immense wards of San Spirito are disgustingly dirty and wretchedly ventilated—and, what is worse, they have the most unscientific, *outrageous* arrangement of "*stowing away*" the poor patients in *double tiers—two tiers* on each side of the ward; the heads of patients in tier No. 2, lying at the feet of those in tier No. 1. I have never seen so bad an arrangement in any hospital—nor one so calculated to produce bad effects. More is the shame, too, for this hospital is *very* richly endowed. The Foundling Hospital and the Lunatic Asylum are also in this building, which, as you may imagine, is immensely large. In the lunatic department the old restraint system is still in use. There are several other hospitals in Rome; indeed, it is the boast there, "that no city in the world devotes so large a sum to institutions of charity, in proportion to the population." But some master's hand is wanted to direct and apply the abundant means. La Consolazione, near the Capitol, is the hospital allotted to *surgical* cases—a good number of these are *stabbing* cases. It is stated that the average number of patients is about 800 annually.

I attempted to enter the Hospital of San Michele, which is very large, *twice*, but was prevented each time: once because it was the "*sleeping time;*" the other visit, on account of its being fête day. The exclusion of visiters at the time when patients are asleep is certainly a good idea: and I have often thought that the visits in the Parisian hospitals, made at so early an hour, are decidedly more for the advantage and convenience of the physician and student than that of the *poor patient*, who is often roused from a slumber of great *importance to him*, to respond to the interrogatories of the visiter. Certainly, on the score of comfort and likelihood of benefit to *patients*, the visiting hour as it is with us is far preferable.

San Michele is highly spoken of, and is doubtless worthy of the praise. It contains a House of Industry and of Correction. It is to be trusted that it is cleaner than San Spirito.

Florence, whose admirable and very extensive collection of anatomical models in wax is so well known to all medical travellers, and indeed *universally* visited, contains, I believe, only two or three hospitals. One of these, Santa Maria Nuova, is worthy of all praise for the remarkably excellent management exhibited. It is the medical school of Florence, and contained, at the time I saw it, 600 patients, having accommodations for 400 more. The cabinet of pathological and anatomical specimens, although small, contained many very good pieces: the skeleton of a child, with the bones of the skull pushed widely apart by hydrocephalic effusion, the head being enormous—I believe larger than any one I have seen; many specimens of excessive distortion of the spinal column; some wax models of tumors, &c. &c. In a small cabinet are preserved the pieces of the human body *petrified* by *Segato*. There were portions of the liver, the brain, the intestines; also the organs of animals. You doubtless have heard of the *table-top*, inlaid with petrified pieces of this nature; it, also, is kept in this cabinet.

The hospital is remarkably airy, neat, well arranged, and has an air of great comfort; the different attendants are exceedingly polite, and every part of the hospital was shown with great readiness, and *pride*, too, as I thought. In the midwifery department are many separate rooms, in which the beds were very clean—the nurses neat, and looking quite good-natured. There is a room for delivery, and others for those affected with after troubles. In this department was shown to me a bed different in construction from any I happen to have seen; it has, about one-third of the way from its head, a slight elevation (continued, of course, *to* the head;) beneath the pelvis an aperture sufficiently large for the issue of matters from the genital organs; not large enough to interfere with the proper support of the body. Besides these things, there is a *succession of cushions*, to regulate, at pleasure, the position of the woman, and two cranks or handles (moveable or fixed, at pleasure,) by which the woman supports herself while undergoing the contractile efforts of the womb. If I remember right, the elevated portion at the head of the bed did not admit of graduation; I may be mistaken in this, however, as it would seem that it *should* and might easily.

The splendor of some of the buildings now devoted to hospitals in Italy is quite striking. In point of architecture, and, often, internal decoration, there probably is nothing of the same destination that equals them.

At Venice, the building known as the Scuola di San Marco is now a portion of an *immense* hospital, the remainder of which is formed out of the adjoining convent of the Dominican and the Franciscan friars. It is really a *long walk* through this enormous building, which contains a handsome church and a smaller chapel also within its walls. The arrangement and planning of the wards, beds and appurtenances, is most excellent—plenty of air, and evidently great attention to cleanliness. The lunatic wards are in excellent condition —and so, as to comfort and attention, are the unfortunate inmates. This, I believe, was one of the first institutions to abandon the restraint system. We entered the large room which contains the greater number of lunatics. Very many were working in one way or another; some came and gazed upon us, with not an *idiot*, but a *mischievous leer*, (this was the female ward;) only one was at all violent. She rushed towards us from the farther extremity of the hall, uttering a torrent of words in a loud tone, and brandishing her *knitting work!* I looked sharp at the *needles*, but she, on arriving where we stood, stopped a moment, and then darted to a seat, where she continued scolding. But the physician of the establishment soon entered, and going to her, took her hands in his, and saying a few words she was quiet as if by magic. Some two or three more were *wild*, but not violent; every part of this establishment seems admirably managed.

In the sick wards there is suspended over the head of each bed the name of the disease under which the individual is laboring.* I noticed a goodly crop of *bronchitis*, many cases of pericarditis, several also of intermittent fever: in the surgical wards one case of spina bifida; abscess, fracture, &c. At the head of each bed also hangs a ticket, containing name, profession, age, dates, symptoms, internal and external remedies, &c. &c.

I should *think* that more medicine was given internally in the Italian hospitals than in the French—a supposition which may have some slight confirmation in the different *size* of the *spaces* allotted to *external* and *internal* remedies in the ticket alluded to. A very formidable, but exceedingly good-looking, apothecary's shop is located, sentinel-like, near the entrance to the wards. The remark above made in reference to the finish and ornament of some of the Italian hospitals, applies in its full extent in this case: an author, speaking of the building, says—"The external architecture of its elevation is singularly fanciful and elegant; Byzantine richness blending itself with the grace of classical architecture, combinations defying all rules, but productive of a most magical effect. The carved work of the ceilings is, in many of the rooms, peculiarly beautiful; the contrast and effect are singular and striking in glancing from the rich and varied ornaments above and around, to the pallid countenances and paraphernalia of the sufferers stretched beneath."

At Milan, the "Ospedale Maggiore" is a noble establishment; a *donation* of the site of an ancient palace by Francesco Sforza in 1456 was its commencement. The front is 800 feet in length; the writer of Murray's Guide Book speaks of the Gothic portion of the building as "magnificent." Besides this fine institution, there are the Lazaretto and the Ospizio Trivulzi, the latter styled by the same writer a "noble monument of pious charity," containing 600 inmates, all over 70 years of age, well fed and clothed, and permitted once a

* This I have not noticed elsewhere in Italy.

week to visit their friends. The Lazaretto, now disused, except in some portions for small shops, is in the form of a square cloister, *one quarter of a mile* on each side in length; in the central square is a chapel. A fine crop of hay had been made, and lay spread upon the turf; men and women in some parts of the square still turning it. The long, cloistered arcades, are quite striking. The Trivulzi I have not visited.

At Genoa, the great Poor House is well worth seeing; clean, and well administered; its church, Santa Maria, contains an invaluable work of Michael Angelo. Beside this, are the Ospedale del Pammatone, and the Deaf and Dumb Institution; the former again confirming the remark made in regard to the ornate appearance of many of these institutions.

I remain respectfully yours, &c.,

Wm. W. Morland, M. D.

